# Measurement of Cooling Detection Thresholds for Identification of Diabetic Sensorimotor Polyneuropathy in Type 1 Diabetes

**DOI:** 10.1371/journal.pone.0106995

**Published:** 2014-09-12

**Authors:** Zoe Lysy, Leif E. Lovblom, Elise M. Halpern, Mylan Ngo, Eduardo Ng, Andrej Orszag, Ari Breiner, Vera Bril, Bruce A. Perkins

**Affiliations:** 1 Division of Endocrinology and Metabolism, Department of Medicine, University of Toronto, Toronto, Ontario, Canada; 2 Division of Neurology, Department of Medicine, University of Toronto, Toronto, Ontario, Canada; Medical University Innsbruck, Austria

## Abstract

**Objective:**

Compared to recently-studied novel morphological measures, conventional small nerve fiber functional tests have not been systematically studied for identification of diabetic sensorimotor polyneuropathy (DSP). We aimed to determine and compare the diagnostic performance of cooling detection thresholds (CDT) in a cross-sectional type 1 diabetes cohort.

**Research Design and Methods:**

136 subjects with type 1 diabetes and 52 healthy volunteers underwent clinical and electrophysiological examination for DSP classification concomitantly with the Toronto Clinical Neuropathy Score (TCNS) and three small fiber function tests: CDT, heart rate variability (HRV), and laser doppler imaging of axon-mediated neurogenic flare responses to cutaneous heating (LDI_FLARE_). Area under the curve (AUC) and optimal thresholds were determined by receiver operating characteristic (ROC) curves in the type 1 diabetes cohort.

**Results:**

Type 1 diabetes subjects were 42±17 years of age with mean HbA1c 7.9±1.7%. Fifty-nine (45%) met the case definition for DSP. CDT values were lowest in cases with DSP (18.3±8.4°C) compared to controls without DSP (28.4±3.5°C) and to healthy volunteers (29.6±1.8°C; p-value for both comparisons<0.0001). AUC_CDT_ was 0.863 which was similar to AUC_TCNS_ (0.858, p = 0.24) and AUC_HRV_ (0.788, p = 0.05), but exceeded AUC_LDIFLARE_ (0.750, p = 0.001). The threshold of <25.1°C was equivalent to the lower bound of the healthy volunteer 95% distribution [25.1, 30.8°C] and performed with 83% sensitivity and 82% specificity.

**Conclusions:**

Akin to novel small fiber morphological measures, CDT is a functional test that identifies DSP with very good diagnostic performance. These findings support further research that revisits the role of CDT in early DSP detection.

## Introduction

Diabetic sensorimotor polyneuropathy (DSP) represents the most common complication of diabetes and predisposes to neuropathic pain, sensory and autonomic dysfunction and ultimately to sequelae such as limb amputation.[1]–[3] Lifetime ulceration risk is estimated at 25%, and such ulcers are themselves associated with exaggerated risk of future amputation.[4], [5] Identification of DSP at early stages may facilitate the selection of subjects for disease-modifying clinical trials and for targeted interventions in clinical practice. Such identification can be accomplished by simple physical examination maneuvers such as vibration sensation testing with the 128 Hz tuning fork or a protective sensation with the 10 g monofilament, but substantial concern exists over the precision, the applicability, and the protocol adherence of physical examination tests in clinical practice.[6]–[9]

Tests for abnormality in small fiber function are appealing for use as early DSP screening tests because the prevailing hypothesis on the pathophysiology of neuropathy is that the initial damage to small, unmyelinated or thinly myelinated Aδ and C-type nerve fibers precedes large fiber damage.[10]–[12] Small fiber damage may be perceived as pain and disturbance in thermal perception whereas large fiber damage is characterized by impairment in vibration, proprioception and protective sensation – modalities that have generally been recommended for neuropathy screening in diabetes.[6], [13], [14] These observations justify aggressive and systematic evaluation of objective and quantitative measures of small nerve fiber function.

The most commonly used small nerve fiber function test in specialized neuropathy assessment is quantitative sensory testing using cooling detection threshold (CDT) measurement which evaluates the perception of cold stimulus.[15]–[17] Though traditionally included as an ancillary test in specialized peripheral nerve function laboratories, CDT has fallen out of favour as a single screening test for diabetic neuropathy for concerns over reproducibility and cost.[17] Though exploration of new methods for detection of small fiber morphological injury is a major focus in current clinical research for diabetic neuropathy,[13], [18]–[24] traditional and novel functional measures such as heart rate variability (HRV) and laser Doppler imaging of the axon-mediated neurogenic flare in response to cutaneous heating (LDI_FLARE_) have been considered.[21], [25]

The concurrent validity of CDT for the identification of DSP has not been previously evaluated in a type 1 diabetes cohort. Nested within the baseline examinations in the Toronto Neuropathy Cohort,[21] we sought to describe the distribution of CDT in healthy volunteers and determine whether it could identify clinical and preclinical stages of DSP in type 1 diabetes subjects.

## Research Design and Methods

### Subjects and Ethics Statement

We examined 136 subjects with type 1 diabetes and 52 healthy volunteers from an ongoing longitudinal cohort (JDRF grant No. 17-2008-715) evaluating the concurrent and predictive validity of small nerve fiber measures. The protocol and consent procedures were conducted in accordance with the World Medical Association's Helsinki Declaration and were approved by the Multidisciplinary Research Ethics Board of the Toronto General Hospital Research Institute. All participants provided written informed consent. Type 1 diabetes subjects were accrued using the Toronto Clinical Neuropathy Score (TCNS), a validated grading assessment that uses elements of history and physical examination to estimate severity of neuropathy.[26] This accrual method was used in order to obtain subjects with a large spectrum of nerve injury, from a lack of injury to severe DSP, and in doing so avoid spectrum bias in the selection of the study population. Using sample size calculations described previously,[21] 20 subjects per category of nerve injury (absent, mild, moderate and severe) were required assuming α-level of 0.05 and 95% power. Healthy volunteers were recruited from friends and family of the diabetes subjects, and from community advertising, and were included in this study with the ancillary goal of describing the distribution of CDT in a healthy population.

Comprehensive medical and neurological examinations were conducted on each subject and involved assessment of neuropathy-related symptoms and signs (blood pressure, heart rate), lifestyle factors, comorbidities (including smoking), biochemical tests (HbA1c, serum lipids, urinary albumin excretion), and tests of small and large nerve fiber function. Type 1 diabetes subjects were included if they were ≥ 18 years of age, had a diagnosis of diabetes as defined by the 2008 (and 2013) Canadian Diabetes Association Guidelines and the ability to understand and cooperate with study procedures.[27] Subjects were excluded if they had confirmed neuropathy owing to non-diabetic causes (familial, alcoholic, nutritional, uremic), and healthy volunteers were excluded if they presented with HbA1c of 5.7 to 6.4%, in keeping with the American Diabetes Association definition for pre-diabetes.[28] No healthy volunteers accrued had HbA1c ≥6.5% consistent with the diagnosis of type 2 diabetes.[28]

### Assessment of small nerve fiber function

CDT, a quantitative sensory threshold test, was assessed using the Medoc device (TSA-II NeuroSensory Analyzer, Medoc, Israel). A stimulator was applied to the dorsum of the foot at a temperature of 32°C and gradually decreased to the first level detected by the patient as cooler than the preceding level. Subjects depressed a button when they perceived the cooling sensation and the sensory threshold was recorded. The test was performed five times bilaterally on the great toe. The five trials from each foot were averaged to establish mean CDT thresholds and compared to age-matched normative data; a catch trial involving a null stimulus was inserted between the five trials at random to ensure patient understanding of the procedure.

HRV was measured using the Dantec Keypoint Workstation (Natus Medical, San Carlos, CA) according to a defined protocol described elsewhere.[29] Briefly, an electrocardiogram tracing was obtained by placing two surface electrodes on the chest. A baseline was recorded at rest followed by R-R interval variation (RRvar) over 1 minute of deep breathing.

Axon reflex-mediated neurogenic vasodilation in response to cutaneous heating by the LDI_FLARE_ technique was measured using the MoorLDI2 (Moor Instruments Ltd, Axminster, UK).[30] A 44°C heating probe was applied to the skin on the dorsum of the right foot for 20 minutes. Blood flow in the dermal capillaries was measured over a 6×6 cm area using MoorLDI software (version 3.11) [30] and the LDI_FLARE_ area was calculated in centimeters squared.

### Classification of DSP case and control subjects

Type 1 diabetes subjects were stratified into three subgroups: clinical DSP cases (the reference standard), preclinical DSP cases (an outcome for exploratory analysis), and controls without DSP. Clinical DSP cases were defined according to published clinical and electrophysiological criteria.[31], [32] In brief, clinical cases presented with a nerve conduction abnormality in both the sural sensory nerve and peroneal motor nerve, in addition to at least one clinical sign or symptom. Signs included abnormal knee or ankle reflexes, temperature, light touch, pinprick, and vibration, and symptoms included numbness, tingling, weakness, foot pain, and ataxia. As a way to classify early-stage DSP, we defined an exploratory “preclinical DSP” class. Preclinical DSP cases were defined as having at least one nerve conduction abnormality in the sural or peroneal nerve without meeting full clinical case criteria – implying that a preclinical case could present with a single abnormal nerve conduction result, with or without signs or symptoms. Controls without DSP had normal nerve conduction results for every one of the five parameters tested. Nerve conduction studies were performed on the sural and peroneal nerve distributions of the left lower limb using the Sierra Wave instrument (Cadwell Laboratories Inc., Kennewick, WA).[18] Parameters measured included sural nerve amplitude potential and conduction velocity, and peroneal nerve amplitude potential, conduction velocity, and F-wave latency.[21], [33]

### Statistical analysis

Statistical analysis was performed using SAS version 9.3 for Windows (SAS Institute, Cary, NC) and R statistical software (version 2.10.1). Baseline clinical characteristics are reported for each variable as either mean ± standard deviation, frequency and percent, or median and interquartile range, as indicated in Table 1. Baseline categorical variables were compared across each of the healthy volunteer and type 1 diabetes subgroups using logistic regression, while continuous variables were compared using ANOVA (for parametric distributions) or the Kruskal-Wallis test (for non-parametric distributions). Individual subgroup comparisons of CDT were made using the Wilcoxon rank-sum test. Linear associations between CDT and other variables were explored through linear correlation; because of CDT's negative-skewed distribution, these associations were assessed using Spearman rank-correlations. Receiver operating characteristic (ROC) curves used to identify DSP were generated for CDT, LDI_FLARE_, HRV, and the TCNS. In order to test the ability of CDT and the other measures to detect either late- or early-stage disease, ROC curves were generated using two methods: one used clinical DSP as the gold-standard for disease identification, while the other used preclinical DSP. A statistical comparison of each tests' area under the curve (AUC) was performed using the method of Pencina.[34] The optimal operating thresholds for each test were determined by finding the point on the ROC curve closest to the top-left corner of the graph (which represents perfect discrimination), according to the distance equation 
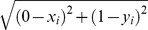
. P-values less than 0.05 were considered statistically significant.

**Table 1:** Characteristics of 52 healthy volunteers and 136 subjects with type 1 diabetes.

		Type 1 Diabetes			
		(N = 136)			
	Healthy Volunteers (N = 52)	Controls without DSP (n = 37)	Preclinical DSP Cases (n = 40)	Clinical DSP Cases (n = 59)	ANOVA P-value
*Clinical characteristics*					
Female sex, n(%)	26(50)	17(46)	25(63)	29(49)	0.47
Age (yr)	35±14	30±13	40±16	50±14	<0.0001
Diabetes Duration (yr)	-	13±8	21±14	31±14	<0.0001
Smoking, n(%)	10(19%)	4(11)	10(25)	7(12)	0.31
Body Mass Index (kg/m^2^)	24.9±5.1	23.8±2.3	25.6±4.4	27.5±5.2	0.003
Systolic Blood Pressure (mmHg)	123±14	125±14	124±15	136±18	<0.0001
Diastolic Blood Pressure (mmHg)	75±9	69±8	71±8	73±9	0.003
Resting Heart Rate (bpm)	69±10	65±13	70±13	72±13	0.07
TCNS, median[IQR]	0[0, 2]	2[0, 4]	2[0, 6]	9[6], [12]	<0.0001
*Biochemical measurements*					
HbA1c (%)	5.4±0.2	7.6±1.2	7.5±1.4	8.5±2.0	<0.0001
Total Cholesterol (mmol/L)	4.63±0.96	4.47±0.88	4.62±0.76	4.5±1.44	0.90
LDL Cholesterol (mmol/L)	2.71±0.68	2.45±0.66	2.58±0.73	2.3±1.03	0.16
Triglycerides (mmol/L)	0.93±0.45	0.94±0.65	0.8±0.49	1.1±0.81	0.23
Creatinine (µmol/L)	72±14	76±14	71±14	86±32	0.01
Urine ACR (mg/mmol)	0.5[0.4, 0.8]	0.8[0.4, 1.2]	0.5[0.3, 1.0]	1.1[0.6, 4.1]	0.005
eGFR (ml/min/1.73 m^2^)	93±16	93±14	89±15	75±24	<0.0001
*Large fiber measures*					
Sural nerve amplitude potential (µV)	19.2±8.2	14.5±4.6	8.9±4.1	2.9±2.2	<0.0001
Sural nerve conduction velocity (m/s)	51.6±4.3	47.3±4.0	45.8±4.9	38.9±4.1	<0.0001
Peroneal nerve amplitude potential (mV)	6.6±2.2	7.0±2.0	4.9±1.9	2.3±1.7	<0.0001
Peroneal nerve conduction velocity (m/s)	48.3±3.2	45±2.5	42.9±2.5	35.8±5.6	<0.0001
Peroneal nerve f-wave latency (ms)	47.2±6.8	49.8±4.1	55.2±9.6	66.9±10.6	<0.0001
*Functional small fiber measurements*					
Heart Rate Variability (%)	38±21	42±21	42±24	22±16	<0.0001
LDI_FLARE_ area (cm^2^)	3.48±1.82	2.28±1.39	2.40±1.19	1.45±0.72	<0.0001
Cooling Detection Threshold (°C)	29.0±1.8	28.4±3.5	25.8±6.8	18.3±8.4	<0.0001

Data presented as mean ± sd and/or median[IQR], unless otherwise noted. P-values for comparison are from the ANOVA test (for continuous parametric variables), the Kruskal-Wallis test (for continuous non-parametric variables), or from logistic regression (for dichotomous variables).

DSP, diabetic sensory polyneuropathy; TCNS, Toronto clinical neuropathy score; HbA1c, glycated hemoglobin; LDL, low density lipoprotein; ACR, albumin-to-creatinine ratio from spot urine samples; eGFR, estimated glomerular filtration rate; LDI_FLARE_, axon–reflex mediated neurogenic vasodilatation in response to cutaneous heating by the laser doppler imaging flare technique.

To address concerns over the effects of age and body mass index (BMI) on thermal thresholds,[35] the diagnostic performance of CDT was also evaluated using methods accommodating covariates. This was explored in two ways: first, covariate-adjusted ROC curves for CDT in the identification of clinical DSP were generated using the method of Janes and Pepe.[36] Additionally, multivariate logistic regression models were generated in order to compare their ability to discriminate between cases and controls. These models used clinical DSP as the dependent outcome variable, and age, BMI, and CDT as the independent predictor variables. Model discrimination was measured by AUC.

## Results

Characteristics of the 52 healthy volunteers and 136 type 1 diabetes subjects are presented in Table 1. The mean age of the healthy volunteers was 35±14. Mean age and diabetes duration of the type 1 diabetes subjects was 42±17 years and 23±15 years, respectively. Among the subjects with type 1 diabetes, 37(27%) were classified as controls without DSP, 40(30%) had preclinical DSP, while 59(43%) had clinical DSP. The prevalence of clinical DSP observed in this cohort was comparable to previous reports.[37] All subgroups had similar gender distributions, smoking prevalence, and lipid profiles. Age, diabetes duration, BMI, systolic blood pressure, TCNS, and HbA1c were all higher for clinical DSP cases compared to all other subgroups, as indicated in Table 1. Incrementally lower values for all large fiber measures was observed across the categories from left to right in Table 1 (p<0.0001 for all parameters). For the functional small fiber measures, HRV was lowest amongst the clinical DSP cases (p<0.0001), while LDI_FLARE_ generally showed incrementally lower values across the categories from left to right in Table 1. CDT showed a similar incrementally lower threshold value across these categories, but the lowest values were seen primarily in clinical DSP cases as compared to all other subgroups including the healthy volunteers.

**Figure 1:**
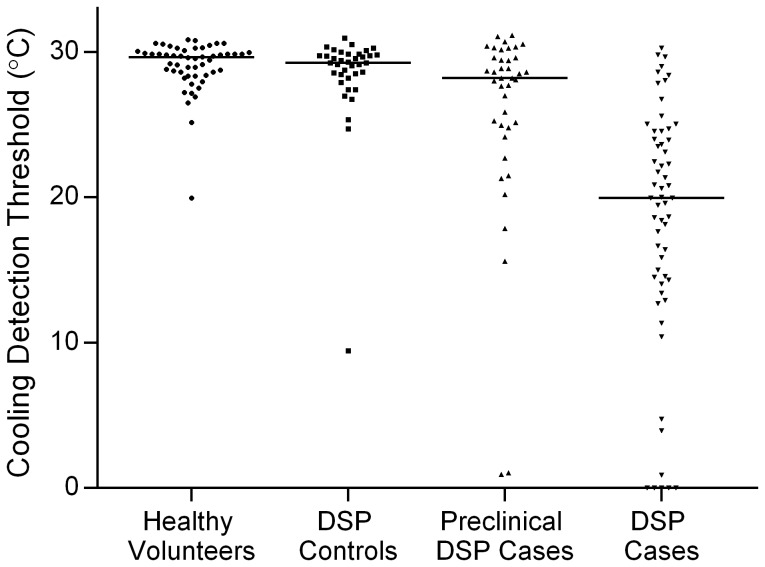
Distribution of CDT values according to DSP case-control status in the 52 healthy volunteers and 136 subjects with type 1 diabetes. Horizontal bars represent the median for each group.

Figure 1 shows the detailed distribution of CDT values according to case-control categories. Levels were highest in healthy volunteers with a mean of 29.0±1.8°C and median[IQR] of 29.6°C [28.5, 30.0°C]. The 95% distribution in healthy volunteers was 25.1°C to 30.8°C. CDT was incrementally lower between the type 1 diabetes subgroups: controls without DSP, preclinical DSP cases, and clinical DSP cases had mean values of 28.4±3.5, 25.8±6.8, and 18.2±8.4°C, respectively; and median values of 29.3 [28.5, 29.8], 28.2 [24.9, 29.6], and 20.0°C [14.3, 24.6], respectively (p<0.0001 for trend). Individual subgroup comparisons showed a significant difference in CDT between clinical DSP cases and both preclinical DSP cases and controls without DSP (p<0.0001 for both comparisons). The CDT for preclinical DSP cases and DSP controls were similar (p = 0.05). The CDT for healthy volunteers was similar to controls without DSP (p = 0.28) but was significantly higher than both preclinical DSP cases and clinical DSP cases (p<0.01).

Results of a linear correlation analysis demonstrate that in healthy volunteers higher CDT was associated with lower TCNS (Spearman rank-correlation r = −0.39, p = 0.004), more normal large fiber measures including sural nerve amplitude potential (r = 0.35, p = 0.01) and conduction velocity (r = 0.38, p = 0.005), peroneal nerve amplitude potential (r = 0.37, p = 0.007), conduction velocity (r = 0.32, p = 0.02), and f-wave latency (r = −0.40, p = 0.003), and the small fiber measure LDI_FLARE_ (r = 0.41, p = 0.002). Pertinent negative associations were observed for two potential confounder variables: the Spearman rank-correlation for age was −0.24 (p = 0.09) and for BMI was −0.08 (p = 0.61). Among type 1 diabetes subjects, more impaired (lower) CDT was associated with older age (r = 0.52, p<0.001), longer diabetes duration (r = 0.49, P<0.001), higher BMI (r = 0.20, p = 0.04), higher systolic blood pressure (r = 0.28, p = 0.001), and higher TCNS (r = 0.58, p<0.0001). Among the diabetes subjects, as observed in healthy volunteers, lower CDT was also associated with more abnormal large fiber measures including sural nerve amplitude potential (r = 0.65, p<0.0001) and conduction velocity (r = 0.64, p<0.0001), and peroneal nerve amplitude potential (r = 0.51, p<0.0001), conduction velocity (r = 0.66, p<0.0001), and f-wave latency (r = −0.59, p<0.0001). Furthermore, lower CDT levels were associated with more abnormal small fiber measures including lower LDI_FLARE_ (r = 0.36, p<0.0001) and lower HRV (r = 0.51, p<0.0001).

**Figure 2:**
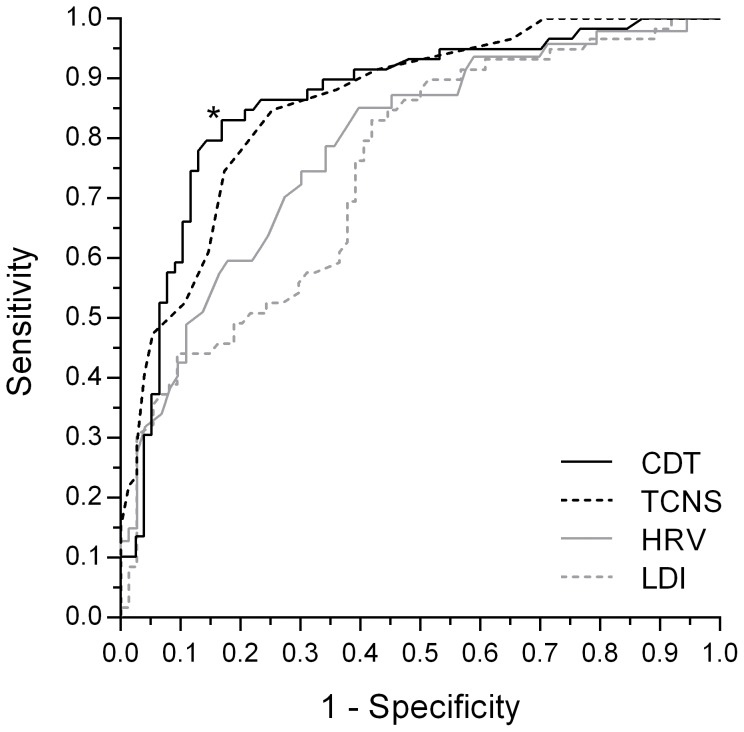
ROC curves for functional small fiber measures and the TCNS in the identification of clinical DSP in 136 Subjects with type 1 diabetes. Clinical DSP was defined as having a nerve conduction abnormality in both the sural sensory nerve and peroneal motor nerve, in addition to at least one clinical sign or symptom. AUCs for CDT, TCNS, HRV, and LDI were 0.863, 0.858, 0.788, and 0.745, respectively. The optimal threshold for CDT (*) was 25.1°C (83% sensitivity, 82% specificity).

ROC curves for the identification of clinical DSP by CDT and other small nerve fiber function tests in the type 1 diabetes cohort are shown in Figure 2. For reference, Figure 2 also shows the ROC curve for the full clinical evaluation as represented by TCNS. Among the four tests, AUC_CDT_ was the highest at 0.863. Compared to AUC_CDT_, AUC_TCNS_ was 0.858 (p = 0.24) and AUC_HRV_ was 0.788 (p = 0.05). However, AUC_LDIFLARE_ was significantly lower than CDT (0.750, p = 0.001). CDT had the best operating characteristics of all measures. This corresponded to an optimal threshold value of 25.1°C, indicating that CDT values <25.1°C were associated with 83% sensitivity and 82% specificity for the identification of DSP cases. Positive and negative predictive values were 79% and 86%, respectively. Positive and negative likelihood ratios were 4.9 and 0.2, respectively. The optimal threshold value of 25.1°C for identification of DSP among diabetes subjects was identical to the lower bound of the 95% distribution of CDT values in the healthy volunteers.

In the exploratory ROC analysis using preclinical DSP as the outcome measure, each of the small fiber function tests and TCNS showed similar AUC for identifying preclinical DSP, when compared to the results seen for clinical DSP. Specifically, AUC_CDT_ was highest at 0.800; AUC_TCNS_ was 0.741 (p = 0.20 for comparison with AUC_CDT_), AUC_HRV_ was 0.692 (p<0.0001), and AUC_LDIFLARE_ 0.618 (p<0.0001). CDT had the best operating characteristics, corresponding to an optimal threshold value for preclinical DSP of <28.5°C, which had 76% sensitivity and 76% specificity for the identification of preclinical DSP cases. Positive and negative predictive values were 89% and 54%, respectively. Positive and negative likelihood ratios were 3.1 and 0.3, respectively.

The covariate-adjusted ROC curves for CDT in the identification of clinical DSP yielded high AUC: the age-adjusted ROC curve had AUC of 0.834, and the age- and BMI-adjusted ROC curve had AUC of 0.788. The multivariate logistic regression model using clinical DSP as the outcome (dependent) variable, and age & BMI the predictor (independent) variables yielded an AUC of 0.849, while the model including CDT as an additional predictor variable yielded an AUC of 0.898.

## Discussion

We performed a cross-sectional analysis in a cohort of subjects with type 1 diabetes with a broad spectrum of nerve injury to determine the concurrent validity of CDT for identification of DSP and we described the distribution of values within a healthy volunteer cohort. In type 1 diabetes, an optimal CDT threshold value of <25.1°C was associated with very good operating characteristics (83% sensitivity, 82% specificity) for the identification of clinical DSP, it was quantitatively associated with DSP severity, and it was identical to the lower bound of the 95% distribution in healthy volunteers. In context, the single measure of CDT had diagnostic performance comparable to an extensive clinical evaluation (the TCNS score), to quantitative measures of small fiber function (HRV and LDI_FLARE_), and even to measures of small fiber morphology[21] for the identification of DSP defined by gold standard electrophysiology-based criteria. In sensitivity analysis CDT also revealed association – but limited operating characteristics - with a preclinical definition of DSP, and extensive analysis excluded a major confounding effect of age and BMI. Finally, in subjects with type 1 diabetes, impaired levels of CDT were quantitatively associated with biologically relevant risk factors for neuropathy, which included older age, longer diabetes duration, higher BMI, and higher systolic blood pressures, as well as with both small and large fiber measures of sensorimotor polyneuropathy.

The hypothesis on the pathophysiology and progression of DSP in subjects with diabetes is that nerve fiber damage is progressive and affects small thinly myelinated Aδ and unmyelinated C fibers at the onset.[6], [11] By virtue of this hypothesis, there may exist a subset of subjects who have established evidence of nerve damage prior to onset of clinically relevant symptoms or complications of neuropathy, and that the use of small fiber measures might have the greatest sensitivity for identification of this subclinical phase. However, screening tools used in clinical practice – such as screening for deficiencies in vibration, light-touch, and protective pressure sensation – primarily represent the large nerve fiber dysfunction seen at later stages of DSP. These measurements, according to the prevailing concept on progression of neuropathy, are likely to be impaired only following damage to small nerve fibers.[6], [12], [13], [22], [24]

In the current study we found CDT levels to be substantially lower in DSP cases compared to healthy volunteers, as well as compared to subjects with diabetes who did not have DSP. Furthermore, among those without DSP, those that had subclinical evidence of neuropathy as indicated by large fiber abnormality in at least one nerve conduction parameter had lower CDT values compared to those without any such large fiber abnormality. The ROC analysis suggested that CDT performed better at identifying established clinical DSP cases than it did identifying preclinical cases. This observation also applied to the other functional small fiber tests HRV and LDI_FLARE_, though we note that a definition of preclinical DSP has not been validated and our definition was therefore considered an exploratory analysis. Additionally, the association of CDT with nerve conduction study results suggests, as in previous studies [16], that CDT may represent aspects of both small and large fiber damage. It must be emphasized, though, that the diagnostic performance of CDT in the current study for concurrent validity – the cross-sectional identification of DSP cases – far exceeded that of the other functional small fiber tests.

Despite the very good concurrent validity of CDT for identification of the presence of clinical DSP, there is a major need for subclinical measures of DSP that can predict the future onset of neuropathy. This is a major priority for the design of future clinical trials that are needed to evaluate putative disease-modifying interventions in patients at high future risk of neuropathy, as well as a priority for identifying such high-risk patients in clinical practice in order to further modify their risk factors for neuropathy, such as further intensification of glycemic control.[37]–[40] Though a quantitative score derived from examination with the Semmes-Weinstein monofilament was able to discern a range of values that indicated risk of future neuropathy onset in a previous study,[8], [41] major concern has been raised over the applicability and reproducibility of physical examination maneuvers in clinical practice,[9] making the use of a quantitative sensory test such as CDT attractive in this setting. CDT is easy to perform, requires minimal training to administer and interpret and it could potentially be implemented as an outpatient tool in diabetes clinics.[16] Though its role for detecting earlier stages of neuropathy is in question, the potential use of CDT in clinical practice needs to be considered in future research. The exploratory analysis used to determine CDT's effectiveness in identifying early, preclinical stages of DSP was done in a cross-sectional manner using an un-validated definition of early disease. Proper evaluation of CDT as a measure of preclinical disease able to predict the future onset of DSP will require a longitudinal study rather than the approach used in our current analysis.

Although the current analysis was well-designed to evaluate the concurrent validity of CDT, it has limitations. First, it was cross-sectional and therefore conclusions about predictive validity can only be inferred from associations seen with preclinical DSP: validation will require longitudinal study. Second, this study was limited to subjects with type 1 diabetes and healthy volunteers. As such, generalizability of the diagnostic performance of CDT in subjects with type 2 diabetes, for whom small fiber damage may be more prevalent early in their natural history,[6], [13], [22]–[24] will require further study. Third, we did not compare measures of small fiber function to the gold standard morphological test – intraepidermal nerve fiber density.[18], [19], [21] Finally, this analysis was not designed to assess the cost-effectiveness of a general practice screening program using CDT as an individual method for neuropathy identification.

We conclude that CDT is a traditional functional small fiber test that in a contemporary, systematic cross-sectional analysis of patients with type 1 diabetes identifies DSP with very good diagnostic performance, even akin to the cross-sectional performance of novel small fiber morphological measures such as in vivo corneal confocal microscopy.[21] These findings support future clinical research into early detection of DSP using CDT in longitudinal cohort designs.
